# A multicenter, randomized, double-blind, placebo-controlled trial to test efficacy and safety of transcranial direct current stimulation to the motor cortex after stroke (NETS): study protocol

**DOI:** 10.1186/s42466-022-00171-2

**Published:** 2022-04-18

**Authors:** The NETS Trial Collaboration Group, Christian Gerloff, Kirstin-Friederike Heise, Robert Schulz, Friedhelm C. Hummel, Silke Wolf, Antonia Zapf, Diana Cordes, Christian Gerloff, Kirstin-Friederike Heise, Friedhelm Hummel, Robert Schulz, Silke Wolf, Kerstin Haevernick, Heike Krüger, Linda Krause, Anna Suling, Karl Wegscheider, Antonia Zapf, Jürgen Dressnandt, Barbara Schäpers, Christoph Schrödl, Björn Hauptmann, Anja Kirchner, Anna Brault, Alexander Gutschalk, Constanze Richter, Dennis A. Nowak, Jitka Veldema, Giacomo Koch, Michele Maiella, Christian Dohle, Katrin Jettkowski, Mario Pilz, Farsin Hamzei, Lydia Olischer, Caroline Renner, Marcus Groß, Michael Jöbges, Bernhard Voller

**Affiliations:** 1 Hamburg, Germany; 2 Department of Neurology, University Medical Center Hamburg-Eppendorf, 20246 Hamburg, Germany; 3 Department of Health Sciences and Research, College of Health Professions, Medical University of South Carolina, 77 President Street, MSC 700, Charleston, SC 29425 USA; 4 Department of Movement Sciences, KU Leuven, 3001 Leuven, Belgium; 5 Defitech Chair of Clinical Neuroengineering, Centre for Neuroprosthetics (CNP) and Brain Mind Institute (BMI), Swiss Federal Institute of Technology (EPFL), Campus Biotech, 1202 Geneva, Switzerland; 6 Defitech Chair of Clinical Neuroengineering, CNP and BMI, Swiss Federal Institute of Technology Valais (EPFL Valais), Clinic Romande de Readaptation (CRR), 1951 Sion, Switzerland; 7 Clinical Neuroscience, University Medical School of Geneva (HUG), 1202 Geneva, CH Switzerland; 8 Department of Medical Biometry and Epidemiology, University Medical Center Hamburg-Eppendorf, 20246 Hamburg, Germany

**Keywords:** Stroke, Brain stimulation, Neuroplasticity, Recovery, Clinical trial, Therapy, Neurorehabilitation

## Abstract

**Introduction:**

The WHO estimates that each year 5 million people are left permanently disabled after stroke. Adjuvant treatments to promote the effects of rehabilitation are urgently needed. Cortical excitability and neuroplasticity can be enhanced by non-invasive brain stimulation but evidence from sufficiently powered, randomized controlled multi-center clinical trials is absent.

**Methods:**

Neuroregeneration enhanced by transcranial direct current stimulation (tDCS) in stroke (NETS) tested efficacy and safety of anodal tDCS to the primary motor cortex of the lesioned hemisphere in the subacute phase (day 5–45) after cerebral ischemia. Stimulation was combined with standardized rehabilitative training and repeatedly applied in 10 sessions over a period of 2 weeks in a planned sample of 120 patients. Primary outcome parameter was upper-extremity function at the end of the 2-weeks intervention period of active treatment or placebo (1:1 randomization), measured by the upper-extremity Fugl-Meyer assessment. Sustainability of the treatment effect was evaluated by additional follow-up visits after 30 and 90 days. Further secondary endpoints included metrics of arm and hand function, stroke impact scale, and the depression module of the patient health questionnaire.

**Perspective:**

NETS was aimed at providing evidence for an effective and safe adjuvant treatment for patients after stroke.

*Trial registration*: ClinicalTrials.gov Identifier NCT00909714. Registered May 28, 2009.

**Supplementary Information:**

The online version contains supplementary material available at 10.1186/s42466-022-00171-2.

## Introduction

Stroke causes the vast majority of disability-adjusted life years among neurological diseases, > 50% of stroke survivors suffer from long-term deficits. Among these, upper extremity (UE) dysfunction is a major problem.

On hospital admission, 50–80% of stroke patients exhibit UE dysfunction. Validated and responsive scoring instruments to describe upper extremity function include upper extremity Fugl-Meyer assessment (UEFMA), action research arm test (ARAT), nine-hole-peg test (NHPT), box-and-block test (BBT), or simply grip force measured with a dynamometer.

An ischemic stroke lesion is followed by extensive changes of brain metabolism, functional activation, neuronal excitability, and structure. While pattern and extent of plasticity and reorganization depend on the amount and localization of initial tissue damage, some key findings point to target areas which could contribute crucially to the recovery of function. For UE motor function, these areas include primary motor cortex (M1), ventral and dorsal premotor cortex, supplementary motor area, primary somatosensory cortex, and posterior parietal cortex of the lesioned and, to some extent, the contralesional hemisphere [13–15, 20].

Approaches to increase cortical plasticity include (1) excitability-enhancing drugs like serotonin-reuptake inhibitors and (2) excitability-enhancing non-invasive brain stimulation (NIBS) like anodal transcranial DC stimulation (tDCS) or high-frequency repetitive transcranial magnetic stimulation (rTMS). Recent trials on drugs to enhance neurorehabilitation have been disappointing [5]. For NIBS, large-scale, placebo-controlled randomized multicenter trials in the acute or subacute phase after stroke are absent. The NICHE trial studied 1-Hz rTMS to the contralesional M1 in 199 patients 3–12 months after stroke and was neutral [7]. Nevertheless, proof-of-principle trials suggest that NIBS, especially anodal tDCS, could improve sensorimotor function [1, 3, 9, 10].

Sample sizes of available studies range mostly from 4 to 35 patients. A meta-analysis on 29 studies (351 patients) suggested a small but significant effect for tDCS (18 of 29 studies) on fine-motor skill function when applied to patients after stroke (effect size, 0.31;95% CI 0.08–0.55; *P* = 0.01) [17]. However, Begg’s funnel plot was asymmetric and suggested publication bias. The most frequently applied strategy is enhancing M1 excitability in order to facilitate neuronal plasticity [4, 9, 11, 12, 19].

## Methods

### Aim of the trial

To test efficacy and safety of anodal tDCS to the M1 of the lesioned hemisphere, combined with standardized training, over a period of 2 weeks in the subacute phase after ischemic stroke.

### Study description and study design

NETS was an investigator-initiated, interventional, randomized, placebo-controlled, parallel-assignment, international multicenter efficacy and safety study with quadruple blinding (patient, care provider, investigator, outcomes assessor). Patients were randomized 1:1 to treatment (anodal tDCS to M1 of the affected hemisphere) or placebo stimulation. The effect of treatment was assessed by comparing the UEFMA before and after the 2-weeks intervention period. The first study protocol dates back to 2009, several protocol modifications were necessary in the course of the trial. These changes are described in the section ‘Time course of the trial and changes of the study protocol’ below.

#### Patient population and eligibility criteria

Patients with ischemic subcortical or cortical, first clinically overt stroke confirmed by CT scan or MRI were included. Time window of inclusion was 5–45 days after stroke. As the primary interest of the study was to improve UE motor function, patients were included only if they exhibited moderate to moderately severe UE paresis, defined as UEFMA score 20–58 (inclusive) to indicate preserved basal hand function. For detailed inclusion and exclusion criteria see Tables S1 and S2 of the Additional file 1.

#### Clinical assessment

After registration of demographic data and stroke subtype, medical history was taken, followed by physical and neurological examination including the Edinburgh handedness inventory and a mini-mental state examination (MMSE). Clinical variables were collected by trained raters. Stroke severity was expressed as National Institutes of Health stroke scale (NIHSS) score. The Barthel index (BI) was used as a generic measure of independence in activities of daily living (ADL). UE function was quantified by UEFMA, ARAT, NHPT, BBT, muscle strength according to Medical Research Council (MRC), pinch and grip force as measured with a dynamometer. Sensory function was measured with von-Frey monofilaments. Spasticity was assessed by the Ashworth scale. As metrics of health-related quality of life (QoL), the stroke impact scale (SIS) and the short version of the patient health questionnaire were used (PHQ-9). More details on the scores are given in the SOM. All data were stored in an electronic Case Report Form. Monitoring was conducted by the Clinical Trial Centre North (Hamburg, Germany) and in compliance with E6 ICH GCP guideline.

### Arms and interventions

#### Randomization

Eligible patients were randomized to treatment with anodal tDCS or placebo stimulation (1:1). Randomization was stratified by age (< 70 years/ ≥ 70 years) and lesion type (subcortical or strokes also involving the cortex). Randomization was performed web-based using centre-wise block stratification with variable block size.

#### Investigational product

**Fig. 1:**
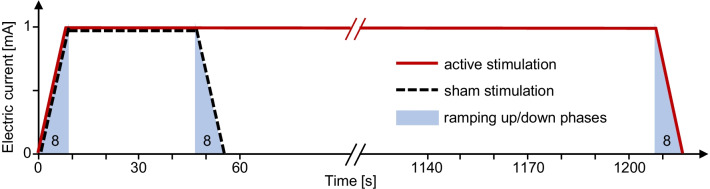
Schematic of active tDCS (solid red line) and placebo stimulation (dashed black line). For both active and placebo stimulation, current was ramped up over 8 s to 1 mA (blue shaded areas). Active stimulation remained at this level for 1200 s (20 min) followed by fade-out over 8 s from 1 to 0 mA. Placebo stimulation remained stable at 1 mA for only 40 s before fade-out over 8 s

For active stimulation, anodal tDCS (1 mA) was delivered for 20 min through 35 cm^2^ (5 cm × 7 cm) sponge-electrodes soaked with sodium-chloride solution leading to a current density of 0.03 mA/cm^2^ (Eldith, Neuroconn, Germany). The anode was centered at C3/4 of the international 10/20 system of EEG electrode placement (left/right hemisphere, depending on stroke location), near the hand representation area of the M1. This approach was deemed to be feasible for routine use and is sufficiently accurate given the large area of the stimulation electrodes used for tDCS. This electrode montage had been applied previously and had exerted reliable and durable effects on M1 excitability [8, 16] as well as some behavioral effects in smaller studies on chronic stroke patients [1, 9]. The cathode was placed over the contralateral supraorbital region. Current was applied with an 8 s fade-in and fade-out interval to attenuate itching sensations. For the placebo condition, anodal tDCS was limited to 40 s, a procedure demonstrated to warrant successful blinding [2, 6] (Fig. 1).

#### Treatment

**Fig. 2:**
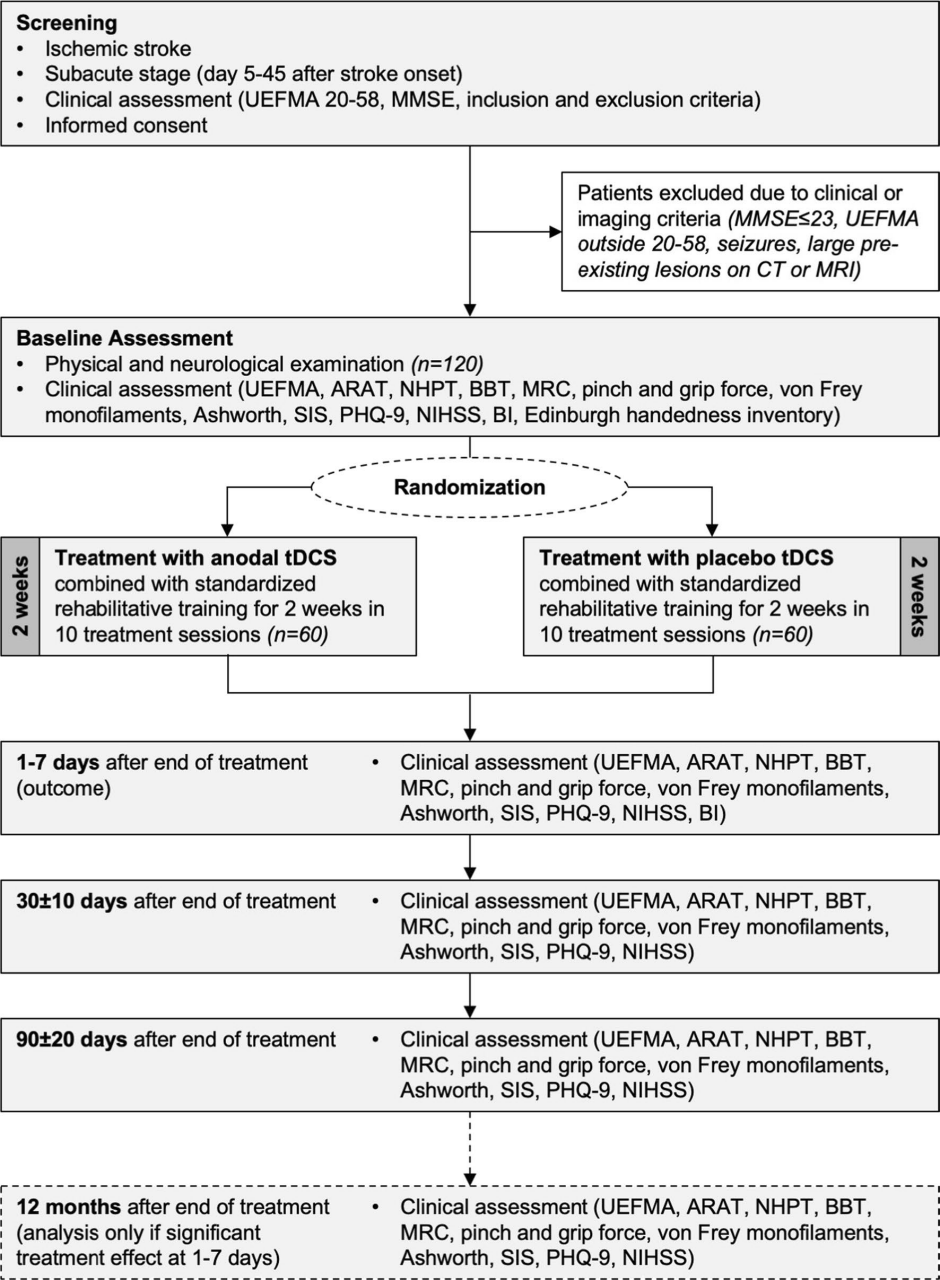
Assessment flow chart. In addition, at each time point a physical and neurological examination took place

Active anodal tDCS (20 min) and placebo tDCS were applied once every workday over 2 weeks (10 working days) in combination with 45 min standardized rehabilitative training of UE function. Training started with onset of brain stimulation so that both treatments were given concurrently for 20 min in the active stimulation condition (40 s for placebo). In both conditions, the stimulator indicated after 20 min that the electrical intervention was completed. The electrodes could then be removed and the training session continued. The contents of rehabilitative training were described and illustrated in detail in a manual. All therapists and investigators participated in specific training modules by the central NETS team. These modules comprised (1) standardized rehabilitative training, (2) application of tDCS, and (3) assessment of primary and secondary outcome measures. After placement of electrodes, tDCS was applied by entering a code and thereby initiating a pre-set, masked program on the stimulator (respective code was obtained in the randomization procedure). Thus, patients, therapists, care takers and evaluators of outcome measures were blinded to the type of intervention. There was no formal requirement that participants needed to be inpatients or outpatients. See Fig. 2 for illustration of assessments.

### Outcome measures

#### Primary endpoint

UEFMA 1–7 days after end of intervention.

#### Primary safety endpoint

Incidence of epileptic seizures during intervention period.

#### Secondary endpoints

Secondary endpoints included UEFMA 30 ± 10 days and 90 ± 20 days after intervention as well as ARAT, NHPT, BBT, muscle strength, pinch and grip force, evaluations of sensory function, spasticity, QoL and ADL at days 1–7, 30 ± 10, and 90 ± 20 after intervention. For more details see Additional file 1.

#### Statistical analysis

NETS was planned to show superiority of the experimental intervention compared with placebo in an intention-to-treat (ITT) population. The ITT population consists of all patients randomized who received at least one session of active or placebo stimulation. The per-protocol population (PP) includes all patients randomized who have no major protocol violation (e.g., < 9 of 10 stimulations applied or violation of inclusion or exclusion criteria). The primary analysis is based on an ANCOVA model with the difference of first post-intervention UEFMA (day 1–7 after end of treatment) to baseline UEFMA as response variable, treatment group and type of stroke as factors, and baseline UEFMA, age, and time interval between index event and baseline as covariates. The treatment effect is judged by the estimated contrast between treatment groups. Secondary endpoints are evaluated analogously to primary outcome measure. No adjustment for multiplicity is provided since these analyses are explorative. Descriptive statistics are provided for safety outcomes. In patients lost to follow-up, their last observation is carried forward (LOCF) in order to accomplish the ITT analysis. Adjusted analyses of the primary endpoint are based on the covariate severity of stroke as measured by the baseline NIHSS score. Pre-planned subgroup analyses include (1) subcortical stroke versus stroke involving cortex, (2) younger versus older patients (< median age versus ≥ median age of study population), (3) male vs. female, (4) mild versus moderate and severe stroke (NIHSS < 5 versus ≥ 5), (5) mild versus moderate and severe UE dysfunction (UEFMA ≥ 43 versus UEFMA < 43), and (6) smoker versus non-smoker.

#### Sample size calculation

Sample size calculation was performed using PASS 2008. Given a probability for a type-one error of 5%, an effective sample size of 100 patients per group was initially considered necessary in order to detect a clinically relevant difference of 5 points in the UEFMA [18] with an expected SD of 12.5 UEFMA points and a power of 80% with a two-sample, two-sided t-test. An early drop-out rate of 20% was expected, so the planned sample size was increased by 25%, leading to a cohort of n = 250.

Blinded re-assessment of the sample size was originally planned after 80% of the patients have been recruited or if cessation of funding before completion of recruitment could be anticipated. Because of slow recruitment, this analysis was carried out after inclusion of 76 patients (August 2018) and revealed a residual variance of 67.8, corresponding to a SD of 8.2 rather than the initially assumed 12.5 points of UEFMA based on literature. Hence, we were able to assume that even a sample size of 2 × 40 patients (80 complete data sets) would provide us with a statistical power > 80% to detect a 5-point difference in UEFMA. Based on this but also considering drop-outs and potentially incomplete data sets, the final sample size was set to n = 120.

### Data safety monitoring

An independent data safety monitoring board (DSMB; see Additional file 1) was informed about adverse events and trial progress. Adverse events were adjudicated by two trialists (CG, SW). The trial would have been stopped if there had been a medically relevant increase in major, unexpected adverse events (such as seizures) with anodal tDCS compared with placebo stimulation.

### Study organization and funding

NETS was funded by the Deutsche Forschungsgemeinschaft (GE 844/4-1) and conducted in 11 recruiting centers in 3 European countries (ClinicalTrials.gov Identifier NCT00909714).

### Time course of the trial and changes of the study protocol

NETS was approved by the local ethics committee in February 2009. The first patient was randomized on November 17, 2009. Here, we report the final version of the protocol which is also the basis of the main analysis. NETS has faced multiple challenges causing slow recruitment. Several measures were taken in order to improve recruitment, which resulted in a series of protocol changes during the ongoing trial. The most prominent changes were (1) reduction of sample size to 120 patients (based on interim blinded re-assessment of residual variance); (2) extension of time window after stroke from 21 to 45 days (allowing inclusion of more patients in rehabilitation centers); (3) changing the time point of primary outcome evaluation from 12 months to right after the end of intervention (in order to reduce the number of patients lost to follow-up). The full history of protocol changes is available at https://clinicaltrials.gov/ct2/history/NCT00909714.

## Perspective

NETS is an investigator-initiated, interventional, randomized, placebo-controlled, multicenter efficacy and safety study with quadruple blinding. It adds clinically relevant information to the topic of adjuvant non-invasive brain stimulation after stroke to enhance motor recovery.

## Supplementary Information

**Additional file 1.** Study Details.

## Data Availability

After publication of the trial results the data shall be made available on request.

## Abbreviations

ADL: Activities of daily living
ARAT: Action research arm test
BBT: Box-and-block test
BI: Barthel index
DSMB: Data safety monitoring board
ITT: Intention-to-treat
LOCF: Last observation is carried forward
M1: Primary motor cortex
MMSE: Mini-mental state examination
MRC: Medical Research Council
NETS: Neuroregeneration enhanced by tDCS in stroke
NHPT: Nine-hole-peg test
NIBS: Non-invasive brain stimulation
NIHSS: National Institutes of Health stroke scale
PHQ-9: Patient health questionnaire
PP: Per-protocol
QoL: Quality of life
rTMS: Repetitive transcranial magnetic stimulation
SIS: Stroke impact scale
SOM: Supplementary online material
tDCS: Transcranial direct current stimulation
UE: Upper-extremity
UEFMA: Upper-extremity Fugl-Meyer assessment

## Declarations

### Authors' information

See see Additional file 1 for a full description of The NETS Trial Collaboration Group.
