# Supplementary material for: A multicenter, randomized, double-blind, placebo-controlled trial to test efficacy and safety of transcranial direct current stimulation to the motor cortex after stroke (NETS): study protocol
Source: Neurol Res Pract. 2022 Apr 18;4:14. doi: 10.1186/s42466-022-00171-2 (PMC9014609; doi:10.1186/s42466-022-00171-2)
Supplement: Supplementary file 1 — Additional file 1. Study Details. [file 42466_2022_171_MOESM1_ESM.docx]

# **Additional file 1**

**A multicenter, randomized, double-blind, placebo-controlled trial to test efficacy and safety of transcranial direct current stimulation to the motor cortex after stroke (NETS) – study protocol**

The NETS Trial Collaboration Group*

Corresponding authors:

Christian Gerloff – [gerloff@uke.de](mailto:gerloff@uke.de)

Friedhelm C. Hummel - [friedhelm.hummel@epfl.ch](mailto:friedhelm.hummel@epfl.ch)

**Complete list of Writing Committee Members and Investigators and Declaration of their potential Conflicts of Interest**

*Writing committee in alphabetical order (current affiliations):

Christian Gerloff (Department of Neurology, University Medical Center Hamburg-Eppendorf, 20246 Hamburg, Germany)

Kirstin-Friederike Heise (Department of Health Sciences and Research, College of Health Professions, Medical University of South Carolina, 77 President Street, MSC 700, Charleston SC 29425, USA; Department of Movement Sciences, KU Leuven, 3001 Leuven, Belgium)

Friedhelm Hummel (Defitech Chair of Clinical Neuroengineering, Centre for Neuroprosthetics (CNP) and Brain Mind Institute (BMI), Swiss Federal Institute of Technology (EPFL), Campus Biotech, 1202 Geneva, Switzerland; Defitech Chair of Clinical Neuroengineering, CNP and BMI, Swiss Federal Institute of Technology Valais (EPFL Valais), Clinic Romande de Readaptation (CRR), 1951 Sion, Switzerland) and Clinical Neuroscience, University Medical School of Geneva (HUG), 1202 Geneva, CH)

Robert Schulz (Department of Neurology, University Medical Center Hamburg-Eppendorf, 20246 Hamburg, Germany)

Silke Wolf (Department of Neurology, University Medical Center Hamburg-Eppendorf, 20246 Hamburg, Germany)

Antonia Zapf (Department of Medical Biometry and Epidemiology, University Medical Center Hamburg-Eppendorf, 20246 Hamburg, Germany)

*Investigators in alphabetical order (affiliation according to recruiting site):

Diana Cordes, Christian Gerloff, Kirstin-Friederike Heise, Friedhelm Hummel, Robert Schulz, Silke Wolf (Department of Neurology, University Medical Center Hamburg Eppendorf, 20246 Hamburg, Germany); Kerstin Haevernick, Heike Krüger (Department of Physiotherapy, University Medical Center Hamburg Eppendorf, 20246 Hamburg, Germany); Linda Krause, Anna Suling, Karl Wegscheider, Antonia Zapf (Department of Medical Biometry and Epidemiology, University Medical Center Hamburg-Eppendorf, 20246 Hamburg, Germany); Jürgen Dressnandt, Barbara Schäpers, Christoph Schrödl (Neurologische Klinik Bad Aibling, 83043 Bad Aibling, Germany); Björn Hauptmann, Anja Kirchner (Neurologisches Zentrum Segeberger Kliniken, 23795 Bad Segeberg, Germany); Anna Brault, Alexander Gutschalk (Department of Neurology, University Hospital Heidelberg, 69120 Heidelberg, Germany), Constanze Richter (Kliniken Schmieder Heidelberg, 69117 Heidelberg, Germany); Dennis A. Nowak, Jitka Veldema (Vamed Klinik Kipfenberg, 85110 Kipfenberg, Germany); Giacomo Koch, Michele Maiella (Dipartimento di Neurologia, Fondazione Santa Lucia, 00179 Rome, Italy); Christian Dohle, Katrin Jettkowski, Mario Pilz (Median Klinik Berlin-Kladow, 14089 Berlin, Germany); Farsin Hamzei (Neurologie Moritzklinik, 07639 Bad Klosterlausnitz, Germany); Lydia Olischer, Caroline Renner (NRZ Leipzig, 04828 Bennewitz, Germany); Marcus Groß, Michael Jöbges (Brandenburgklinik Berlin-Brandenburg, 16321 Bernau bei Berlin, Germany); Bernhard Voller (Neurologische Universitätsklinik Wien, 1090 Wien, Austria).

Conflicts of interest:

*Writing Committee (all members are also investigators):*

CG reports (outside the submitted work) funding from European Union, German Federal Ministery of Education and Research (BMBF), German Statutory Pension Insurance Scheme (RV Nord), German National Innovation Fond, Wegener Foundation, and Schilling Foundation; honoraria as speaker or consultant from Abbott, Amgen, Bayer Vital, Bristol-Myers-Squibb, Boehringer Ingelheim, Daiichi-Sankyo, Sanofi Aventis, and Prediction Biosciences.

KFH reports (outside the submitted work) funding from the Research Foundation Flanders (FWO), ACCEL Delaware-CTR an Institutional Development Award (IDeA) from the National Institute of General Medical Sciences of the National Institutes of Health under grant number U54-GM104941 (PI: Hicks).

FCH reports (outside the submitted work) funding from European Union (ERA Net), Swiss National Science Foundation (SNSF), ETH domain (PHRT mechanism), Defitech Foundation, Bertarelli Foundation (Catalyst), Wyss Center for Bio and Neuroengineering.

RS reports (outside the submitted work) funding from Else-Kröner Fresenius-Stiftung, Werner-Otto-Stiftung.

SW reports no conflict of interest.

AZ reports no conflict of interest.

*Investigators:*

DC reports no conflicts of interest.

KH reports no conflicts of interest.

HK reports no conflicts of interest.

LK reports no conflicts of interest.

AS reports no conflicts of interest.

KW reports no conflicts of interest.

JD reports no conflicts of interest.

BS reports (outside the submitted work) funding from European Union, German Federal Ministery of Education and Research (BMBF).

CS reports no conflicts of interest.

BH reports no conflicts of interest.

AK reports no conflicts of interest.

AB reports no conflicts of interest.

AG reports (outside of the submitted work) funding from the Deutsche Forschungsgemeinschaft (DFG).

CoR reports no conflicts of interest.

DAN reports no conflicts of interest.

JV reports no conflicts of interest.

GK reports (outside the submitted work) funding from the Italian Ministry of Health, The Alzheimer’s Drug Discovery Foundation (ADDF), the Brightfocus Foundation.

MM reports no conflicts of interest.

CD reports (outside the submitted work) funding from German Federal Ministery of Education and Research (BMBF); CD reports (outside the submitted work) honoraria as speaker from Pfizer, m & i, SRH, Evangelic Geriatric Centre Berlin; CD reports (outside the submitted work) honoraria fur publication from Schulz-Kirchner Verlag and Hippocampus Verlag.

KJ reports no conflicts of interest.

MP reports no conflicts of interest.

FH reports no conflicts of interest.

LO reports no conflicts of interest.

CaR reports no conflicts of interest.

MG reports no conflicts of interest.

MJ reports (outside the submitted work) funding from European Union, German Federal Ministry of Education and Research (BMBF), German Statutory Pension Insurance Scheme (DRV Bund).

BV reports no conflicts of interest.

**Members of the Data Safety and Monitoring Board (DSMB)**

Prof. Dr. Michael Nitsche (Dept. Psychology and Neurosciences, Leibniz Research Centre for Working Environment and Human Factors, Dortmund, Germany; phone +49 (231) 1084 301; [nitsche@ifado.de](mailto:nitsche@ifado.de))

Prof. Dr. Michael Hennerici (Department of Neurology, University Hospital Mannheim, Medical Faculty Mannheim, University of Heidelberg, Mannheim, Germany; +49 (621) 383 2885; [hennerici@neuro.ma.uni-heidelberg.de](mailto:hennerici@neuro.ma.uni-heidelberg.de))

**Inclusion and Exclusion Criteria**

SOM Table S1 | Inclusion criteria

| - ≥18 years of age - First clinically overt ischemic stroke - Non-hemorrhagic stroke - Subacute stage (5-45 days after stroke onset) at time of inclusion - Subcortical stroke or stroke involving also cortical structures - Moderate to moderately severe upper-extremity hemiparesis, defined as UEFMA 20-58 - Active wrist extension of at least 5 degrees or ability to perform a repetitive grasping task |
| --- |

SOM Table S2 | Exclusion criteria

| - Pre-existing lesions of >1.5 cm (maximum diameter) in a brain area belonging to the anatomically established sensorimotor system - Progressive stroke - Completely lesioned hand-knob area of M1 - Bilateral motor impairment - Present alcohol and/or drug abuse - Present severe psychiatric illness (e.g., schizophrenia) - Severe language disturbances that prevents the patient to give informed consent or inhibit adequate scoring because of insufficient understanding of scoring introductions - Tumor diseases with a life expectancy <1 year - Increased intracranial pressure - Polyneuropathy and/or ischemic peripheral disease if the sensorimotor functions of the upper extremities are affected in a clinically relevant way - Severe cognitive deficits (mini-mental state examination, MMSE, ≤ 23) - Pregnancy - Contraindication for MRI (e.g., metallic implants) or TMS (e.g., epilepsy) |
| --- |

**Secondary Endpoints (complete list and explanations)**

- UEFMA 30±10 days and 90±20 days after the intervention
- Action research arm test (ARAT) 1-7 days, 30±10 days and 90±20 days after the intervention
- Nine-hole peg test (NHPT) 1-7 days, 30±10 days and 90±20 days after the intervention
- Box-and-block Test (BBT) 1-7 days, 30±10 days and 90±20 days after the intervention
- Muscle strength according to British Medical Research Council (MRC) 1-7 days, 30±10 days and 90±20 days after the intervention
- Pinch and grip force (GF) 1-7 days, 30±10 days and 90±20 days after the intervention
- Von Frey monofilament-based assessment of somatosensory function 1-7 days, 30±10 days and 90±20 days after the intervention
- Ashworth spasticity scale 1-7 days, 30±10 days and 90±20 days after the intervention
- Stroke impact scale (SIS) 1-7 days, 30±10 days and 90±20 days after the intervention
- Patient health questionnaire (PHQ-9) 1-7 days, 30±10 days and 90±20 days after the intervention
- National Institutes of Health stroke scale (NIHSS) 1-7 days, 30±10 days and 90±20 days after the intervention
- Barthel index 1-7 days after the intervention
- Section J of the Fugl-Meyer Assessment (passive joint movement and pain) 1-7 days, 30±10 days and 90±20 days after the intervention

All secondary endpoints were also measured at baseline. In addition, all outcome parameters measured on 90±20 days were assessed again at 12 months after the intervention. These measurements are not part of the primary data analysis and will only be analyzed and reported if the primary-endpoint analysis indicates a significant treatment effect.

In the original version of the protocol, we had planned the primary endpoint to be UEFMA 12 months after the end of the intervention. After discussion in the NETS study group, it was decided in June 2017 to change the time point to the assessment directly following the intervention period. There were two main reasons for this change, (i) the variability of treatments and influence of comorbidities between the end of the NETS intervention and the 12-months follow-up examination (which might mask any tDCS effect), and (ii) slow recruitment and the concern that more patients will be lost to a follow-up at 12 months compared with 1-7 days after the intervention, so that the trial might fail because of missing data for the primary endpoint. As a further secondary endpoint, the original protocol had included the Jebsen-Taylor hand function Test, which was later removed because of its duration and a large number of missing data at T0.

**Clinical Scores**

Stroke severity was expressed as National Institutes of Health stroke scale (**NIHSS**) score (0-42, with higher values indicating more severe deficits).

The Barthel index (**BI**) was used as a generic measure of independence in activities of daily living (range 0-100, where 100 indicates full independence).

Upper extremity function was quantified by

- the Fugl-Meyer assessment of the upper extremity (**UEFMA**; range 0-66, with a higher score indicating larger active range of motion),
- the action research arm test (**ARAT**; range 0-57, with a higher score indicating better performance)
- the nine-hole-peg test (**NHPT**; ratio between number of pegs and time required to perform the test [pegs/sec], higher values indicate better performance),
- the box-and-block test (**BBT**; number of blocks moved within 60 sec, higher number indicates better performance),
- muscle strength according to British Medical Research Council (**MRC**; range 0-5, where 5 indicates full strength),
- pinch and grip force of the hand as measured with a dynamometer (in kg).

Sensory function was measured with **von-Frey** monofilaments.

Spasticity of muscle tone was assessed by the **Ashworth** spasticity scale (range 0-4, where 0 indicates no increase in muscle tone).

As metrics of health-related quality of life we used

- the stroke impact scale (**SIS**, summary scores for each of 8 domains amounting to 0-100, where 100 indicates no self-perceived impact on quality of life) and
- the short version of the patient health questionnaire (**PHQ-9**; range 0-27, where 0 indicates the absence of any depressive symptoms).
